# Optimization of Media Change Intervals through Hydrogels Using Mathematical Models

**DOI:** 10.1021/acs.biomac.2c00961

**Published:** 2023-02-01

**Authors:** Floor A.A. Ruiter, Jasia King, Sangita Swapnasrita, Stefan Giselbrecht, Roman Truckenmüller, Vanessa L.S. LaPointe, Matthew B. Baker, Aurélie Carlier

**Affiliations:** † MERLN Institute for Technology-Inspired Regenerative Medicine, Department of Cell Biology−Inspired Tissue Engineering, Maastricht University, P.O. Box 616, 6200 MD Maastricht, the Netherlands; ‡ MERLN Institute for Technology-Inspired Regenerative Medicine, Department of Complex Tissue Regeneration, Maastricht University, P.O. Box 616, 6200 MD Maastricht, the Netherlands; § MERLN Institute for Technology-Inspired Regenerative Medicine, Department of Instructive Biomaterials Engineering, Maastricht University, P.O. Box 616, 6200 MD Maastricht, the Netherlands

## Abstract

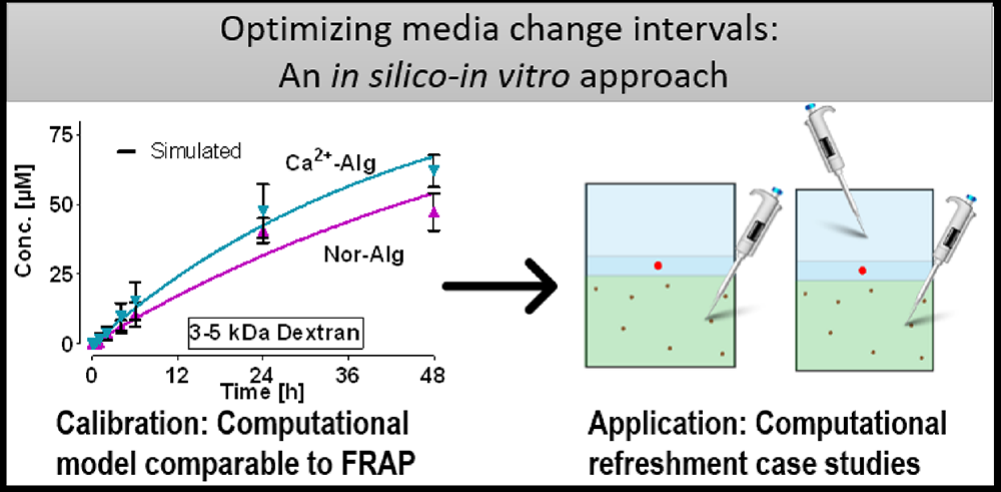

Three-dimensional cell culture in engineered hydrogels is increasingly used in tissue engineering and regenerative medicine. The transfer of nutrients, gases, and waste materials through these hydrogels is of utmost importance for cell viability and response, yet the translation of diffusion coefficients into practical guidelines is not well established. Here, we combined mathematical modeling, fluorescent recovery after photobleaching, and hydrogel diffusion experiments on cell culture inserts to provide a multiscale practical approach for diffusion. We observed a dampening effect of the hydrogel that slowed the response to concentration changes and the creation of a diffusion gradient in the hydrogel by media refreshment. Our designed model combined with measurements provides a practical point of reference for diffusion coefficients in real-world culture conditions, enabling more informed choices on hydrogel culture conditions. This model can be improved in the future to simulate more complicated intrinsic hydrogel properties and study the effects of secondary interactions on the diffusion of analytes through the hydrogel.

## Introduction

Hydrogels are of increasing interest in regenerative medicine due to their similarities to the extracellular matrix in network formation and water content.^1,2^ When cells are encapsulated in hydrogels, diffusion of gases, growth factors, and metabolic waste is of great importance for cell viability and fate.^3^ In the biomaterials field, materials are usually characterized by diffusion coefficients obtained via fluorescent recovery after photobleaching (FRAP) measurements.^4−10^ However, these FRAP measurements focus on structural (mesh size) and local diffusion, while understanding diffusion patterns throughout a bulk hydrogel system is mainly overlooked.^11^ Furthermore, FRAP measurements require expensive and specialized equipment and software to measure the fluorescence intensity of the experiments.

There is a disconnect between information on local diffusion and the practical translation to cell culture. Therefore, we set out to test the ability to couple fluorescence plate reader measurements with mathematical models to improve our understanding of growth factor availability and diffusion across a hydrogel. We were interested to see if the mathematical models can identify the diffusion coefficients of the hydrogel systems as a time- and cost-effective alternative to FRAP. Various mathematical models have been used to investigate the diffusion coefficients of hydrogels in culture as reviewed in refs (12−14) ranging from the traditional hydrodynamic radius defined by the Stokes–Einstein equation at the molecular scale^15^ to multiscale diffusion models that combine the hydrodynamic, free volume, and obstruction theoretical frameworks to capture solute diffusion in, for example, poly(ethylene glycol) (PEG) hydrogels.^16^ More specifically, the hydrodynamic theory considers the friction between the polymer chains of the hydrogel and solute^17^ while the free volume theory assumes that the solute particles move via the dynamic empty spaces between the polymer chains^18^ and the obstruction theory models the hindrance caused by the polymer chains.^19^ These frameworks, in combination with coarse-grained simulations such as Brownian dynamics simulations, have been extended to also include electrostatic interactions^20,21^ and binding^22^ to better capture the dynamic movement of solutes within hydrogel networks. Recently, Amsden systematically compared the predictive quality of four models (i.e., a hydrodynamic model, an obstruction model, an obstruction-exclusion model, and a combined free volume/obstruction model) when either the correlation length or the mesh radius was used as approximation for the mesh size.^23^ Next to these detailed, albeit complex, mathematical models, others have lumped the various interactions into a constant (measured) diffusivity coefficient and used Fick’s law to describe the macroscopic diffusion behavior;^24,25^ most recently, attempts at multi-scale models have started to provide a unified look at the total picture.^16^

**Figure 1:**
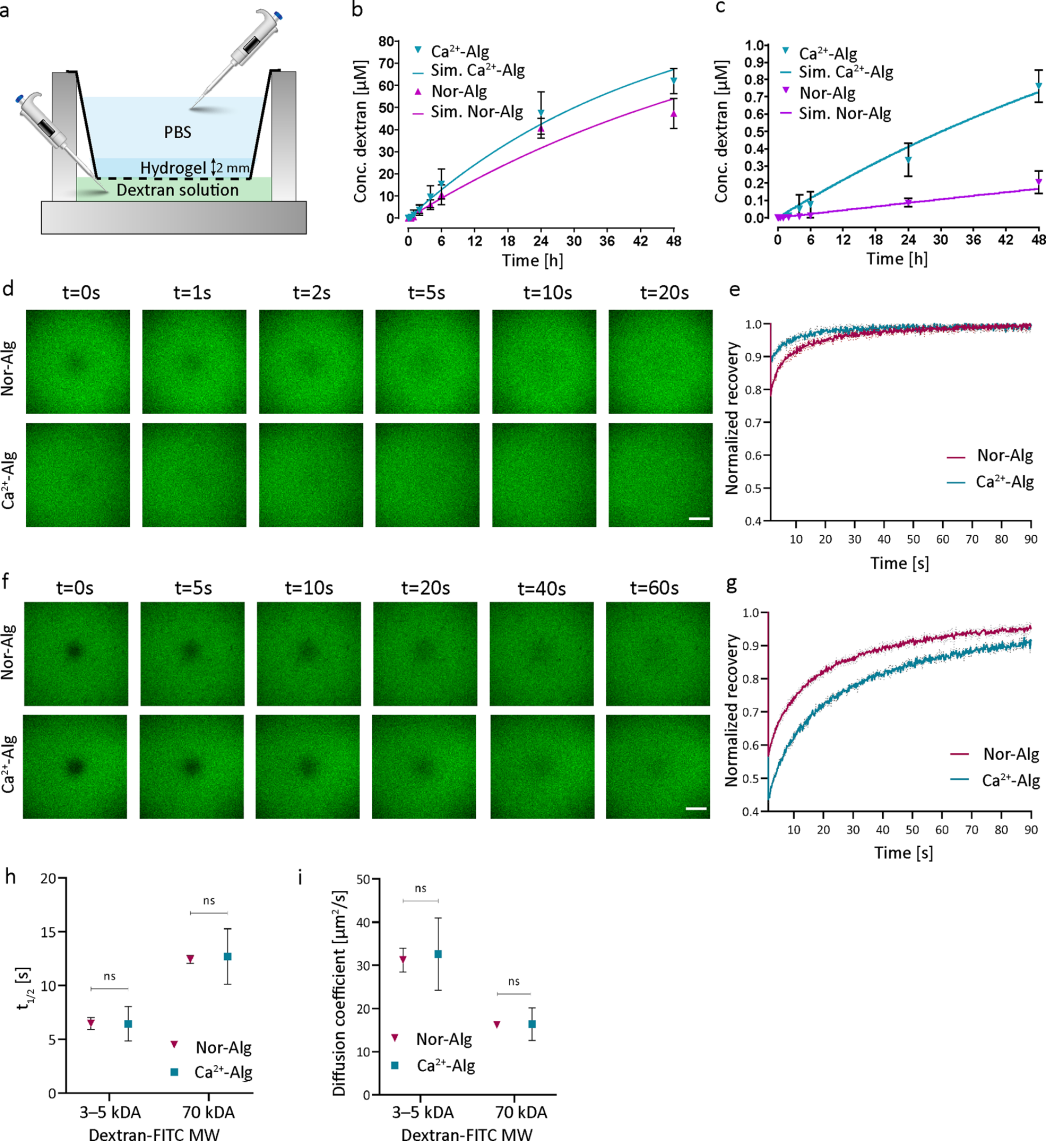
(a) Diffusion time-series data schematic of the experimental setup. Approximately 2 mm-thick hydrogels were cross-linked on a cell culture insert filter, and the diffusion of 3–5 and 70 kDa dextran from the bottom to the top was determined by measuring the concentration in the top compartment for 48 h (*N* = 6 cell culture inserts with hydrogels). Diffusion time-series data, the concentration of (b) 3–5 kDa and (c) 70 kDa dextran in the top compartment after diffusion through the hydrogels (Nor-Alg and Ca^2+^-Alg). A comparison of the experimentally measured diffusion of (b) 3–5 kDa and (c) 70 kDa dextran through Nor-Alg or Ca^2+^-Alg compared to the fitted diffusion coefficient estimation model (solid lines). (d) Fluorescence recovery after photobleaching (FRAP) images of 3–5 kDa dextran from 0 to 60 s in Nor-Alg (top) and Ca^2+^-Alg (bottom) hydrogels, and (e) the corresponding recovery curves (*N* = 3 difference bleaching areas, scale bar:100 μm). f) FRAP images of 70 kDa dextran from 0 to 20 s in Nor-Alg (top) and Ca^2+^-Alg (bottom) hydrogels, and (g) the corresponding recovery curves (*n* = 3 difference bleaching areas, scale bar = 100 μm). (h) The half-time recovery (*t*_1/2_) and (i) diffusion coefficients were not significantly different (ns, one-way ANOVA) for either 3–5 or 70 kDa dextran in Nor-Alg and Ca^2+^-Alg.

Here, we propose an alternative methodology that does not require specialized analytical systems. Instead, we measured the concentration of fluorescently labeled dextran over time (with a multichannel fluorescence plate reader) that passed through a hydrogel of a specific thickness and we developed a diffusion coefficient estimation model of the hydrogel. More specifically, we selected 3–5 and 70 kDa dextran for concentration versus time plots to feed into the model, as many growth factors and nutrients, such as albumin, epidermal growth factor (EGF), fibroblast growth factor (FGF)-2, insulin, and insulin-like growth factor (IGF)-1, have molecular weights of a similar size.^26−28^ Using a cell culture insert, we collected experimental concentration versus time data points (up to 48 h) of 3–5 and 70 kDa dextran diffusing across a 2 mm alginate hydrogel cross-linked in two different ways (thiol-ene; Nor-Alg, 2 wt %, 11.3 μmol norbornene units, cross-linked with 4-arm PEG-thiol and calcium; Ca^2+^-Alg, 2 wt %, 83 mM CaCl_2_, see the experimental details in Dextran Concentration versus Time Data—Diffusion Sample Preparation) (Figure 1a, see Diffusion Measurements of Dextran Concentration Versus Time Data for more details). We designed a comprehensive mathematical model in Virtual Cell, an open-source software, to quantify the diffusion coefficients of 3–5 and 70 kDa dextran by a parameter-fitting algorithm (see Diffusion Coefficient Estimation for more details). The fitted diffusion coefficients were compared with FRAP recovery experimental curves, the gold standard in the hydrogel field. We then developed a COMSOL Multiphysics 5.4 model with the estimated diffusion coefficient to investigate the diffusion of growth factors with a similar molecular weight of 3–5 kDa dextran through the 2 mm hydrogels in relevant cell culture scenarios, such as media refreshments and growth factor degradation. We confirmed that the model reliably simulated cell culture scenarios and highlighted the importance of the growth factor diffusivity of the hydrogels. The controlled and quantified diffusion in hydrogels represents a potent approach to instruct biomimetic development, where controlled signaling gradients instruct cell fate and organoid development, but also to steer differentiation and test the effects of molecules or drugs, requiring immediate signals that can be obtained by controlling the refreshment times of the compartments.

In summary, this study provides proof of concept of a simple, time- and cost-effective alternative to FRAP where fluorescence plate reader measurements are combined with mathematical models to determine the diffusion coefficients in hydrogel systems. In addition, researchers can apply the physical properties of their molecule of interest to the developed models to gain an insight into the best media change practices or the media change intervals that will affect the molecule’s availability in culture, which is particularly important when culturing highly sensitive cells on hydrogels. The proposed integrated approach allows for the quantification and characterization of local concentrations in a variety of cell culture systems, providing a connection between local concentration information and cell culture practice.

## Experimental Section

### Dextran Concentration versus Time Data—Diffusion Sample Preparation

#### Ca^2+^ Cross-Linked Hydrogels

A 2 wt % alginate (30 mg) stock solution of 1.5 mL was prepared. Cell culture inserts (ThinCert cell culture inserts, 8 μm pore size, Greiner Bio-One) were covered by the hydrogel solution to form a hydrogel of approximately 2 mm in height (226.2, μL) by gently spreading the solution evenly with a pipet, after which 1 mL calcium chloride (9.27 g/L, 110.98 g/mol, 83 mM) was added below the cell culture insert left overnight at RT to ensure complete cross-linking. The hydrogel and cell culture insert were washed with PBS to remove any traces of CaCl_2_ before real-time diffusion measurements.

#### Thiol-Ene Cross-Linked Alginate Hydrogels

Norbornene functionalized alginate (71 mg, 11.3 μmol norbornene units) was dissolved in 2.3 mL PBS overnight at RT. The four-arm 10 kDa PEG thiol (5 mg, 0.02 mmol SH units, Creative PEGWorks) and LAP UV initiator (3.3 mg, 11.2 μmol, 3.2 mM, Sigma-Aldrich) were dissolved separately in 1.2 mL PBS. The two solutions were added together to form a 2 wt % norbornene functionalized alginate solution. The dissolve norbornene-alginate and four-arm 5 kDa PEG solution were added to obtain a 2 mm hydrogel on the cell culture inserts. These cell culture inserts with hydrogel solutions were exposed to 365 nm light (10 mW/cm^2^, UVP CL-1000 ultraviolet cross-linker) for 30 s. After cross-linking, cell culture inserts with hydrogels were incubated with 500 μL PBS in the bottom compartment overnight at 4 °C to ensure complete swelling before diffusion measurements.

### Diffusion Measurements of Dextran Concentration versus Time Data

Cell culture inserts with 2 mm-thickness Ca^2+^- or thiol-ene cross-linked alginate hydrogels were transferred into a 12-well plate, and 1 mL of 0.1 mg/mL 70 kDa or 1 mg/mL 3–5 kDa dextran was added in the bottom compartment. The cell culture insert top compartment was topped with PBS (773.8 μL) to obtain a total volume of 1 mL (hydrogel + PBS, 226.2 + 773.8 μL, respectively). Samples (20 μL) from the top and bottom compartments were taken after 5, 15, 30, 45, 60, 120, 240, 260, 1500, and 2940 min and were diluted in 30 μL PBS. The fluorescence intensity was measured at 495 nm excitation and 519 nm emission, and the dextran concentration was calculated from a standard curve (Figure S1f).

### A Computational Model of Diffusion through a Hydrogel

#### Diffusion Coefficient Estimation

A well-mixed compartmental estimation model was developed in the Virtual Cell V7.4.0 software^29,30^ to estimate the diffusion coefficient of the two hydrogels. The estimation model can be found in the VCell Database as “Hydrogel Diffusion Coefficient Estimation” by “aureliecarlier” and can be accessed within VCell software (available at https://vcell.org). The estimation model used real-time data points to fit best the diffusion coefficient of 70 and 3–5 kDa dextran described in the Diffusion Measurements of Dextran Concentration versus Time Data section based on the general first-order Fickian diffusion equation (eq 1):
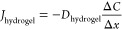
1where *J*_hydrogel_ is the flux of dextran across the hydrogel in micromolar per micrometer per second, *D*_hydrogel_ is the unknown diffusion coefficient of dextran through the hydrogel in square micrometers per second, Δ*C* is the difference between dextran concentrations on either side of the hydrogel in micromolar, and Δ*x* is the thickness of the hydrogel in micrometers.

**Figure 2:**
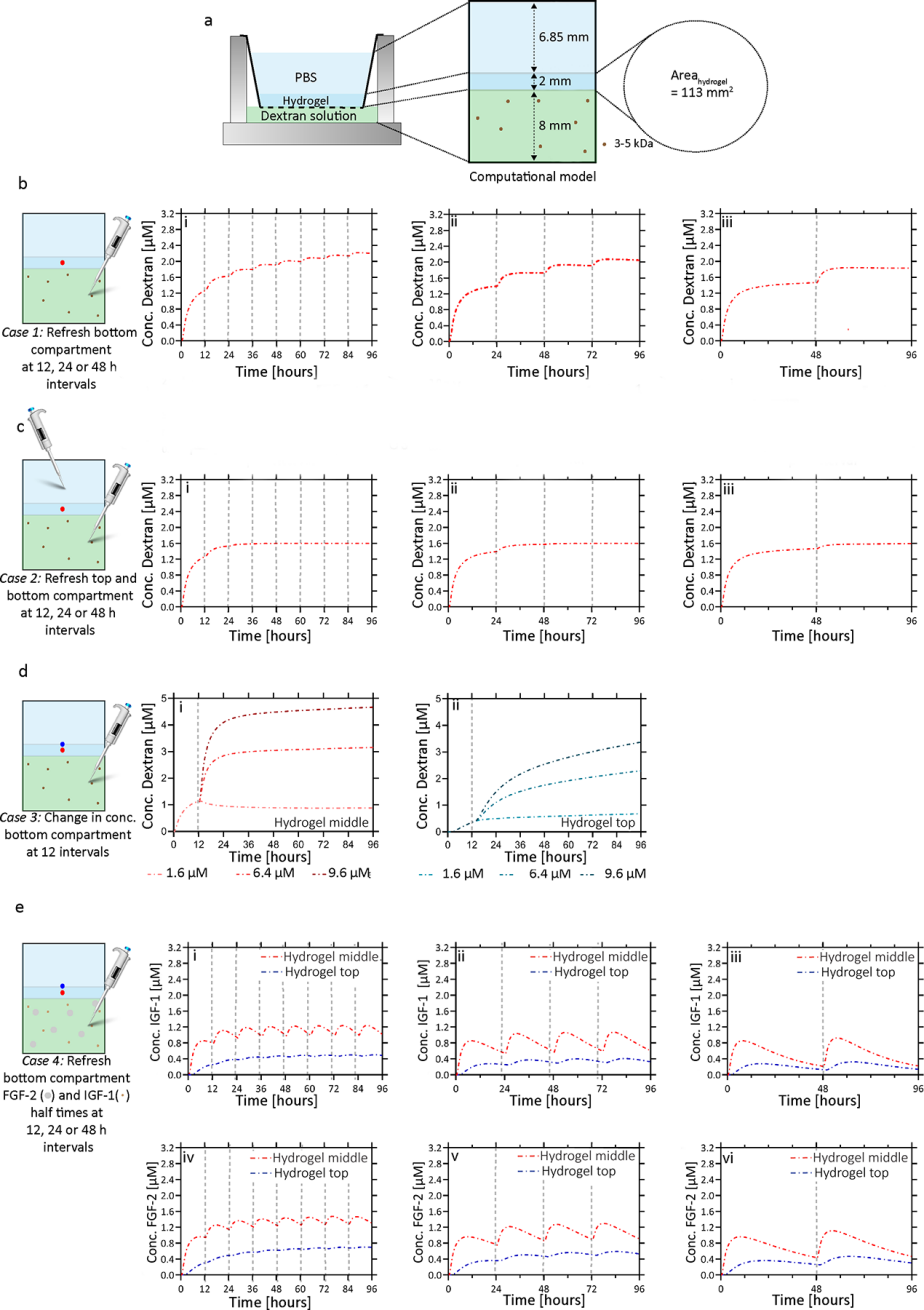
(a) A computational model of growth factor transport in a hydrogel system was designed based on the dextran concentration versus time experimental setup. The height of the bottom compartment is 8.85 mm, the height of the hydrogel is 2 mm, and the height of the top compartment is 6.85 mm. Four case studies were simulated in the Nor-Alg hydrogel system using the diffusion coefficient for 3–5 kDa dextran of 31.3 μm^2^/s. The top and hydrogel compartments had initial concentrations of 0 μM dextran, and the bottom compartment had an initial concentration of 3.2 μM dextran for all cases. (b) Case 1: the concentration (μM) of 3–5 kDa dextran at the middle of a 2 mm-thick Nor-Alg hydrogel was determined over time when the bottom compartment was refreshed with the starting dextran concentration of the bottom compartment (i.e., 3.2 μM) at (i) 12, (ii) 24, and (iii) 48 h. (c) Case 2: the concentration (μM) of 3–5 kDa dextran at the middle of a 2 mm-thick hydrogel was determined over time when the top and bottom compartments were refreshed with the starting dextran concentration of the bottom compartment (i.e., 3.2 μM) at (i) 12, (ii) 24, and (iii) 48 h. (d) Case 3: the concentration (μM) of 3–5 kDa dextran was determined over time when the bottom compartment was refreshed with concentration bursts (1.6, 6.4, and 9.6 μM) of 3–5 kDa dextran after 12 h of 3.2 μM concentration in the (i) the middle of the 2 mm-thick hydrogel (red dot) and (ii) at the top of the 2 mm-thick hydrogel (blue dot). (e) Case 4: the concentration (μM) of IGF-1 (i-ii-iii) with a halftime of 17 h and FGF2 (iv-v-vi) with a halftime of 27 h at the middle (red) and top (blue) of a 2 mm-thick hydrogel as determined over time when the bottom compartment was refreshed at 12, 24, and 48 h with 3.2 μM. Gray vertical striped lines represent media refreshment points. The colors of the lines represent the sampling position. Figure S5 provides additional information on the concentration data for the Nor-Alg simulations at the top and bottom of the hydrogel. Similar results, but for the Ca^2+^-alg hydrogel, can be found in the supplement (Figures S3 and S4).

The 12-well plate Transwell insert parameters were applied to define the domain geometry (Figure 2a) with a bottom compartment volume of 1 mL, top compartment volume of 0.77 mL, hydrogel thickness of 2E3 μm, and area of 1.13E8 μm^2^. Here, the hydrogel was modeled as a boundary condition applied over the Transwell area with a specified thickness Δ*x*, resulting in a flux boundary condition dependent on the unknown diffusion coefficient of the hydrogel. The initial concentration of dextran in the bottom and top compartments was 3.2 and 0 μM, respectively.

An evolutionary programming algorithm was used to fit the real-time data to the model to find the best-fitting diffusion coefficients (*D*_hydrogel_ in eq 1) of 3–5 and 70 kDa using the settings specified in Table 2.

The root mean square error (RMSE) was calculated as shown in eq 2, to evaluate how good the fit was between the average concentration of 3–5 kDa dextran predicted by the estimated diffusion coefficient model and the real-time experimental data at 5, 15, 30, 45, 60, 120, 240, 260, 1500, and 2940 min (Table 2), which correspond to the sampling times of the real-time experimental data points.
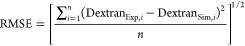
2where Dextran_Exp, *i*_ is the average dextran concentration in the top compartment at time *i* of the real-time experiment in micromolar, Dextran_Sim, *i*_ is the average dextran concentration in the top compartment at time *i* of the simulation in micromolar, *n* is the total number of data points, and *i* is the index of the data points.

More details on developing the computational models of various cell culture scenarios can be found in the Simulating the Diffusion Effects in Various Cell Culture Scenarios section in the Supporting Information.

## Results and Discussion

We started with a diffusion coefficient (first-order Fickian diffusion) estimation using the obtained time-series data of dextran diffusion through the hydrogel (Figure 1a–c). Then, we compared the diffusion through two different cross-linked alginate hydrogel systems: based on (1) a covalent cross-linking via thiol-ene chemistry (Nor-Alg) or (2) an ionic cross-linking of alginate with calcium (Ca^2+^-Alg). The resultant diffusion coefficient estimations from the dextran concentration versus time data were 31 and 15 μm^2^/s for 3–5 and 70 kDa dextran in the Nor-Alg hydrogel, respectively, while the Ca^2+^-Alg hydrogel had diffusion coefficients of 48 and 23 μm^2^/s for 3–5 and 70 kDa dextran, respectively (Table 1). The estimated diffusion coefficients were tested against the time-series data by calculating the RMSE between the experimental and predicted data (Figure 1b,c and Table 2). The simulated time-series data were observed to fit well against the experimental data (Figure 1b,c, solid line), as the calculated RMSE for both fitted diffusion coefficients was less than 15% of the fitted value.

**Table 1:** Fitted Diffusion Coefficient from Dextran Concentration Versus Time Data vs. FRAP Data for 3–5 and 70 kDa in the Two Different Hydrogel Systems (Thiol-Ene Cross-Linked: Nor-Alg and Calcium Cross-Linked: Ca^2+^-Alg)^a^

	thiol-ene cross-linked alginate			calcium cross-linked alginate		
dextran size [kDa]	3–5	70	70	3–5	70	70
FRAP diffusion coefficient (SD) [μm^2^/s]	31.23 (± 2.75)	16.72 (± 0.48)	16.72 (± 0.48)	32.63 (± 8.37)	16.38 (±3.75)	16.38 (±3.75)
Transwell pore size [μm]	0.2	0.2	8	0.2	0.2	8
fitted diffusion coefficient in Transwell (RMSE) [μm^2^/s]	31.30 (3.80)	4.36 (7.8E-4)	15.85 (9.00E-3)	48.39 (2.70)	1.13 (2.20E-2)	23.05 (1.5E-3)

a The Transwell experiments were done for two different Transwell pore sizes resulting in different fitted diffusion coefficients for 70 kDa, whereas FRAP was performed in *N* = 3 individual bleaching areas within the dextran-containing hydrogel.

**Table 2:** COPASI Parameter Fitting Settings for the Evolutionary Programming Solver Chosen to Fit the Diffusion Coefficients

COPASI parameter fitting settings for the evolutionary programming solver		
number of generations	200	number of generations the population evolves
population size	20	number of individuals that survive
seed	1	random number generator
number of runs	10	increased number of runs to check if increasing the number of runs alters the fitted value

COPASI parameter fitting settings for guessing fitted values for the diffusion coefficient estimation			
parameter	initial guess	lower limit	upper limit
*D*_hydrogel_ [μm^2^ s^–1^]	12	1	100

Additionally, the simulated concentration versus time curves were within the standard deviation of their corresponding experimental data points (Figure 1b,c). The accuracy of the diffusion coefficient estimation model was highly sensitive to the initial dextran concentration set in both the top and bottom compartments, since this was when the steepest concentration gradient existed in the model (0 μM in the top versus 3.2 μM in the bottom compartment).

With data from the model in hand, we wanted to utilize FRAP measurements to compare the experimental data of the two hydrogels. Therefore, the FRAP recovery curves for 3–5 and 70 kDa were generated (Figure 1d–g), τ_1/2_ was extracted (Figure 1h), and diffusion coefficients were calculated by the Soumpasis equation (eq S1, Supporting Information; Figure 1i). There was no significant difference in the FRAP diffusion coefficients from Nor-Alg compared to Ca^2+^-Alg hydrogels (one-way ANOVA, *p* = 0.4633). We did notice that the Ca^2+^ alginate samples had slightly different diffusion values between the FRAP and experimental models; this is likely attributed to the known inhomogeneity of CaCl_2_-crosslinked alginates on large scales. While the FRAP data accurately captured the local diffusion coefficients, the large thickness and inhomogeneity of crosslinking across the sample were likely better represented in the Transwell experiment.

We chose to test two cell culture insert pore sizes, namely, 8 μm pore size, as this has large pores that do not interfere with the diffusion measurements, and 0.2 μm pore size, as this is often used in cell culture experiments. The estimated diffusion coefficients from the mathematical model were comparable to those from the FRAP measurements (Table 1) for 3–5 and 70 kDa dextran performed on 8 μm pore size cell culture inserts (Figure 1h,i and Figure S1c). The results showed that the fluorescence plate reader measurements of dextran concentration versus time (taking into account the appropriately sized membrane pore size) can be used to calculate the diffusion coefficients accurately. However, we observed that the 70 kDa diffusion experiments performed on the 0.2 μm pore size cell culture inserts differed significantly from the corresponding FRAP diffusion coefficients (Table 1). We showed that the 0.2 μm pore size resulted in an extra barrier to the diffusion of large nutrients (70 kDa dextran). Indeed, both pore size and dextran molecular weights need to be considered as distributions, resulting in a proportion of dextran to have a hydrodynamic diameter of more than 30 nm and the pores to have sizes around 100 nm. At these values, free diffusion is replaced by pore diffusion, which confounds the measurements. While piecing out this subtlety remains challenging, we can show that the pore size should be carefully considered when designing an experiment with cell culture inserts. Therefore, we used the data obtained from cell culture inserts with the 8 μm pore size in the case studies to follow.

With a calibrated mathematical model in hand, we then sought to quantify the diffusion of growth factors throughout a hydrogel in four cases relevant for cell culture: (1) changing culture media in the bottom cell culture insert compartment, (2) changing media in the bottom cell culture insert compartment and PBS in the top compartment, (3) increasing the media growth factor concentration with a bolus injection of growth factor in the bottom compartment after 12 h, and (4) changing culture media in the bottom compartment including a growth factor decay rate. We designed a mathematical model in COMSOL Multiphysics 5.4 to simulate growth factor diffusion through the 2 mm Nor-Alg (Figure 2 and Figure S5) and Ca^2+^-Alg hydrogels (Figures S3 and S4) using the estimated diffusion coefficients in the 8 μm pore size cell culture insert displayed in Table 1. The diffusion coefficients of the dextran molecules in the media were assumed to be the same as those measured in PBS by FRAP (see data Figure S2).

### Case 1. Media Change in the Bottom Compartment

We modeled the scenario where the media were changed below the hydrogel (bottom compartment) at different time intervals (12, 24, and 48 h) while leaving the top of the hydrogel (top compartment) without refreshment, resulting in the curves presented in Figure 2b. Here, we assumed that growth factors and waste of ±3–5 kDa sizes, such as EGF, FGF-2, insulin, and IGF-1, would diffuse at a similar rate as the dextran diffusion coefficient data obtained (for the results with other diffusion coefficients, see Figure S6). The 3–5 kDa dextran concentration gradient into the hydrogel was observed to decrease at each subsequent media change interval. Here, it was shown that case 2 achieved a steady state after two media changes of both 12 and 24 h intervals. This was considerably faster than case 1, meaning that a researcher should change both the top and bottom compartments if they want to develop a steady-state concentration of growth factors across the hydrogel as fast as possible. This can be needed in systems where immediate signals are relevant, for example for directed differentiation and the testing of drugs or screening of molecules. More specifically, in the first case, when specific signaling pathways need to be induced for specific durations in a cell culture system without a hydrogel, the cells receive these signals immediately as a pulse (upon adding them to the culture medium).^31^ When translating such a system to a hydrogel setup, the diffusion alters the dynamics in which the signals are provided, and in order to be comparable and provide the required pulsed stimuli, the steady-state concentration needs to be established as fast as possible in a hydrogel setup by changing the medium in both top and bottom compartments (case 2). Similarly, when testing or screening drugs or molecules for toxicity in, for example, organoids or aggregates of cells (which are often encapsulated in a hydrogel to improve how well they mimic the tissue), it is important to understand how quickly these drugs/molecules reached the cells and at what dose.^32^

The concentration of growth factors above the hydrogel remained steady, and the final concentration after 96 h in the top compartment was similar for all the media change intervals, approximately 1.3–1.6 μM for 3–5 kDa dextran (Figure S5a). Calculations from the middle of the hydrogel showed increased growth factor concentrations immediately after refreshing the media at all time intervals. However, the hydrogel, acting as a diffusion barrier, reduced the concentration gradient, resulting in a smooth concentration curve measured in the top compartment of the cell culture insert (Figure S5a). The observed plateaus in the concentration curves within the hydrogels in case 1 indicated a dampening capacity of the hydrogel (Figure 2b and Video S1). We use the term “dampening capacity” to define the hydrogel’s ability to dissipate significant disturbances from the compartment below when the medium is changed. Due to the dampening capacity of the hydrogel, the cells cultured on the top of the hydrogel would sense gradual changes in concentration, despite repeated refreshment or bolus injections.

### Case Study 2. Media and PBS Change in the Bottom and Top Compartments

To further explore the dampening capacity of the hydrogels, a new model was simulated to include analyte removal in the top compartment. When case 2 was compared with case 1, it was observed that the dampening property in the middle of the hydrogel (Figure 2b and Figure S3c) occurred in a shorter time frame; i.e., Figure 2b−c shows that the concentration difference between the time intervals was factually zero after the second refreshment in case 2 for all simulated intervals, while case 1 was still establishing its steady state by the last refreshment simulated. This finding shows the potential to create a diffusion gradient in a hydrogel system by simply changing the media or PBS in the bottom and top compartments. Furthermore, this stable diffusion gradient is noticeable when visualizing cases 1 and 2 in two-dimensional space (Video S1), where the dextran concentration in the hydrogel is gradually affected by the sudden media refreshments. Similarly, solute removal (reflecting for example metabolite removal) results in concentration fluctuations in the top and bottom parts of the hydrogel whereas the concentration within the hydrogel reaches a steady state level (see Figure S7). Note that case 2 represents a means to control diffusion gradients with an in vitro hydrogel culture, which is important to instruct biomimetic development.^33^ Indeed, the gradients of growth factors and signaling molecules are extremely important in the culture of more advanced multi-cellular models like organoids, since development is carefully controlled by signaling gradients that control and regulate cell fate in a concentration-dependent manner.

### Case Study 3. Concentration Burst/Bolus Injection

We then simulated how changes in the concentration of the 3–5 kDa growth factors (bolus injections of 1.6, 3.2, 6.4, or 9.4 μM) in the bottom compartment after 12 h would affect the simulated growth factor gradient. As expected, the overall concentration of 3–5 kDa dextran at 24 h post-refreshment (36 h time-point) increased in the top compartments from 1.1 to 3.2 and 4.7 μM in the states of 6.4 or 9.4 μM bolus, respectively (Figure 2d and Figure S3d) and decreased to 0.9 and 0.5 in the middle and top compartments, respectively, for 1.6 μM bolus. In addition, a slight delay in increasing growth factors of 1 h was observed in the middle and top compartments. These results show the possibility of changing the growth factor concentration within the hydrogel without a significant burst by gradually increasing or decreasing the growth factor concentration.

### Case Study 4. Growth Factor Decay

We modeled the diffusion profiles of FGF-2 and IGF-1 as they decay based on their half-life and estimated diffusion coefficient. Since cells are typically cultured on or within the hydrogels, we explored the effect of the growth factor concentration in the middle and top of the hydrogels. Figure 2e and Figure S2e (red and blue curves, respectively) show that upon refreshing the media in the bottom compartment at 12 and 24 h intervals, IGF-1 (Figure 2e, i–iii) reached a minimum concentration of 0.38 and 0.25 μM in the top of the hydrogel, respectively, and therefore was available to the cells. However, when the media were changed every 48 h, the IGF-1 concentration decreased to 0.12 μM before refreshment and was less than 0.2 μM for the last 13 h of culture at the top of the hydrogel. It was seen that cells had significantly slower cell doubling times when treated with 5 ng mL^–1^ (0.7 μM) IGF-1 compared to 20 ng/mL (2.7 μM).^34^ Therefore, IGF-1 availability is insufficient for the cells over an extended culture period when media are changed every 48 h, suggesting that more frequent media changes should be performed when using 2 mm-thick hydrogels. Similarly, FGF-2 (Figure 1e, iv–vi) had minimum concentrations of 0.57, 0.36, and 0.26 μM available to the cells on top of the hydrogel at 12, 24, and 48 h intervals, respectively. These results showed that the frequency of media refreshments should be carefully considered in the experimental design, as each growth factor’s half-life directly affects growth factor diffusion and availability in culture over time. This is exemplified with IGF-1, which has a shorter decay time compared to FGF-2, resulting in less availability in the middle and top of the hydrogel.

Our results show that the dextran concentration versus time data through the hydrogel can be used to approximate the diffusion coefficient of macromolecules diffusing through a hydrogel with a mathematical model. Moreover, the results show that this methodology is a reliable alternative to FRAP experiments and may better take into account gel spatial inhomogeneity. This is especially interesting for research groups without reliable access to confocal microscopy but starting with standard wet lab equipment and reagents.

In our studies, we noticed a profound dampening capacity of the hydrogel system. This result indicates that the hydrogel system can accommodate a limited but stable growth factor concentration in the cell environment compared to a conventional monolayer culture in which the growth factor concentration fluctuates due to refreshment intervals. Moreover, this model showed the potential of maintaining a diffusion gradient throughout a hydrogel by simply refreshing the solution around it. However, over time, the degradation of growth factors needs to be considered when deciding on media change frequency, as there might be too few growth factors available for an extended period before the next media refreshment. Therefore, the frequency of media change or the growth factor concentration of interest can be increased to ensure sufficient growth factor availability in the hydrogel. Mehrian et al. similarly demonstrated that neotissue growth supported by a 3D scaffold in a perfused bioreactor was maximized with a high frequency of media refreshments and increased concentration.^35^ Similarly, this model can be used to optimize scenarios where less frequent media changes are preferred, as the cultured cells rely on the secreted products to proliferate or self-organize.

The observed dampening capacity of the hydrogels can be both an advantage and a disadvantage for cell culture. First, the dampening prevents the cells from experiencing a “growth factor shock/stress” with cell culture media changes.^35^ Instead, the concentration gradually increases to a steady state, which can be maintained when carefully considering the correct media change interval and growth factor decay patterns (simulated in our model). Second, growth factor levels can be increased, without the initial burst observed when cells are cultured in media only by applying a higher concentration of growth factors during media changes. Moreover, we observed a steady gradient within the hydrogel system by simply refreshing the compartments above and below the hydrogel. This can be of interest for research focused on the influence of gradients within a biological system to guide cell behavior such as migration, which to date are obtained through microfluidic devices that establish growth factor gradients by flow^36^ or via porosity changes to facilitate diffusion coefficient gradients within the hydrogel.^37^ Although this gradual concentration change and dampening capacity have their advantages, in some cases, a short burst of specific growth factors is required, for example, in the various differentiation protocols of pluripotent stem cells.^38^

There were assumptions made in designing the mathematical models for this study, leading to potential improvements to be made in the future. In particular, as described in the Diffusion Coefficient Estimation model, the hydrogel was simplified as a flux boundary condition dependent on the thickness and diffusion coefficient of the hydrogel. The spatial components of the hydrogel were simplified to generalized diffusive flux to make the model fitting possible. It is known that, next to diffusing through, proteins can bind to the hydrogel or to the surface receptors of cells present within the hydrogel. For example, Limasale et al. used a combined experimental-computational approach, to understand and predict the local concentration of free and bound vascular endothelial growth factor 165 (VEGF165) in glycosaminoglycan (GAG) networks, including both protein diffusion and GAG binding.^25^ Similar approaches can be taken in the future to account for (un)binding reactions that will affect local concentrations and potential gradients. Considering that an increase in complexity will also increase the calculation time for finding the optimal media refreshment settings, it would be interesting to derive statistical relations that directly link the required (input) constraints to the optimal settings, see for example ref (39). Finally, it would be interesting to explore other hydrogel systems to explore whether the proposed methodology is generic.

## Conclusion

This study provides proof of concept of a simple, time- and cost-effective alternative to FRAP where fluorescence plate reader measurements are combined with mathematical models to determine the diffusion coefficients in hydrogel systems. We presented a mathematical model for fitting the diffusion coefficients of 3–5 and 70 kDa dextran through two different hydrogels with open-source software (Virtual Cell). The obtained diffusion coefficients were comparable to those measured through traditional methods (FRAP). Furthermore, we developed four computational scenarios relevant to typical cell culture practices and can visualize the hydrogels’ dampening effect, where the dampening concentration was tunable based on the refreshment method. We showed the importance of considering the decay rate of essential growth factors, which affects their availability for the cells cultured on top of the hydrogels. We also provided evidence that scientists need to be careful when selecting their hydrogels and cell culture methods, for which our proposed integrated approach can be a valuable tool to quantify and characterize the local nutrient and growth factor concentrations, providing a connection between local concentration information and cell culture practice.

## Supporting Information Available

Methodological details: alginate purification, norbornene-alginate synthesis, FRAP sample preparation, FRAP measurements, statistics, computational modeling information; and supporting figures (PDF)
Videos of the dynamic concentration profiles of case 1 and 2 at different refreshment intervals (ZIP)
