# Supplementary material for: Optimization of Media Change Intervals through Hydrogels Using Mathematical Models
Source: Biomacromolecules. 2023 Feb 1;24(2):604–12. doi: 10.1021/acs.biomac.2c00961 (PMC9930106; doi:10.1021/acs.biomac.2c00961)
Supplement: Supplementary file 1 — bm2c00961_si_001.pdf [file bm2c00961_si_001.pdf]

Optimization of Media Change Intervals Through Hydrogels Using Mathematical Models

Floor A.A. Ruiter, Jasia King, Sangita Swapnasrita, Stefan Giselbrecht, Roman Truckenmüller, Vanessa L.S. LaPointe, Matthew B. Baker, Aurélie Carlier

SUPPORTING INFORMATION

EXPERIMENTAL

Alginate purification

Sodium alginate (Manugel GMB, FMC, lot no. G940200) was dissolved in deionized (DI) water at 1 w/v% (10 g) overnight at 4°C. Activated charcoal (Merck) was added at 0.5 w/v% and left to stir for 3 hours. Subsequently, the alginate/charcoal solution were centrifuged for 10 mins, 7500 rpm at 8°C. The resultant supernatant was filtered twice over Celite 545 (0.02–0.01 mm particle size, Merck) sandwiched between two 11 µm filters (Whatman membrane filters mixed cellulose ester, Merck) and once over Celite 545 sandwiched between two 0.2 µm filters. The resultant product was flash-frozen in liquid N2 and lyophilized.

Norbornene-alginate synthesis

As previously described [1] purified sodium alginate was functionalized with norbornene via the EDC-NHS chemistry. Briefly, purified sodium alginate (0.5 g, 2.5 mmol COOH groups, 1 equiv., Manugel GMB, FMC, lot no. G940200) was dissolved overnight in 50 mL MES buffer (0.1 M MES, 0.3 M NaCl, pH 6.5). 1-(3-(Dimethylamino)propyl)-3-ethylcarboiimide hydrochloride (0.5 g, 2.6 mmol, 1.02 equiv., EDC-HCL; VWR) and NHS-ester (Sigma-

Aldrich) were added and left to stir for 30 min. Subsequently, the pH was adjusted to ±8 (with 5 M NaOH), 5-norbornene-2-methylamine (9.4 x 10-2 g, 0.76 mmol, 0.3 equiv., mixture of isomers, TCI Chemicals) was added and stirred overnight (18 h) at room temperature (RT). The norbornene-functionalized alginate product was purified by dialysis (10 kDa MWCO dialysis tube, Spectra/Por, regenerated cellulose, VWR) in 100 mM, 50 mM, 25 mM, and 0 mM NaCl in MilliQ water (water changes every 10–18 h, dialysis ratio 1:50). The resultant product was flash-frozen in liquid N2, its structure and percentage of norbornene functionalization were confirmed by H1-NMR (3.5% Figure S1d) and MW determined by GPC (Figure S1e), as described in Geuens & Ruiter (2021) [1].

FRAP sample preparation

Ca^{2+} cross-linked hydrogels; 2 wt% purified alginate hydrogel solution in PBS was prepared and stirred overnight at RT. This solution was added to the four chambers of an untreated 35 mm 4 compartment culture disk (300 µL each, VWR 391-0255, Greiner bio-one) and 1 mL calcium chloride (9.27 g/L, 110.98 g/mol, 83 mM) solution was gently added for cross-linking and left for 1 h at RT. After cross-linking, the calcium solution was removed, the hydrogels were washed with PBS, and were incubated in 500 µL of 0.1 mg/mL FITC-labelled dextran (70 or 3–5 kDa, Sigma-Aldrich) overnight at RT in the dark.

Thiol-ene cross-linked alginate hydrogels; Norbornene functionalized alginate (71 mg, 11.3 µmol norbornene units) was dissolved in 2.3 ml PBS overnight at RT. The 4-arm 10 kDa PEG thiol (5 mg, 0.02 mmol SH units, Creative PEGWorks) and LAP UV initiator (3.3 mg, 11.2 µmol, 3.2 mM, Sigma-Aldrich) were dissolved separately in 1.2 ml PBS. The two solutions were added together to form a 2 wt% norbornene functionalized alginate solution. This solution was added to the four chambers of an untreated 35 mm compartment culture disk (300 µL each,

VWR 391-0255, Greiner bio-one) and exposed to 365 nm light (10 mW/cm2, UVP CL-1000 ultraviolet cross-linker) for 30 s. After cross-linking, the hydrogels were incubated in 500 µl of 0.1 mg/mL dextran (70 and 3–5 kDa, Sigma-Aldrich) overnight at RT in the dark.

FRAP measurements

FRAP data were measured on the Leica TCS SP8 STED using LAS X FRAP software. Images were taken at a z-height of 40 µm in the hydrogel at 488 nm, 800 V gain, and a bleaching ROI of 60 µm diameter. Timeframes were 0.223 sec per frame, with 5 frames pre-beach (1.2 sec), 90 frames bleaching (21.2 sec) and 400 frames post-beach (110.5 sec). Recovery curves were gathered from three different areas in the hydrogel. Data were processed in ImageJ, main intensity of the bleaching area; full image and background per timeframe were obtained. These .csv files were run through the FRAPBot program (frapbot.kohze.com), where the half-time of recovery was determined. The diffusion coefficient was calculated by the Soumpasis equation in which, D=diffusion coefficient, r=radius of the bleaching area, and $\tau_{1/2}$=the half-time of recovery.

$$D = 0.224 * \left(\frac{r^2}{\tau_{\frac{1}{2}}}\right) \qquad \text{eq. S1}$$

Statistics

FRAP measurements were performed in triplicate (three different bleaching areas within one hydrogel). Real-time data was performed in triplicate, with three separate hydrogels on transwells. Data was processed, and statistical analysis was performed in GraphPad Prism 8.2.0.

A computational model of diffusion through a hydrogel system

Simulating the diffusion effects in various cell culture scenarios, COMSOL Multiphysics 5.4 was used to predict the diffusion of 3–5 and 70 kDa dextran across the hydrogel in the transwell inserts for four cell culture case studies. Typical cell culture practices inspired the case studies (described below) in relation to media changes and growth factors' molecular properties.

The *Transport of Dilute Solutes Module* of COMSOL Multiphysics 5.4 was applied to the three-compartment model (top/hydrogel/bottom compartment) in two-dimensional space as described in Figure 2A with a bottom compartment volume of 1 mL, top compartment volume of 0.77 mL, hydrogel thickness of 2E3 µm, and area of 1.13E8 µm^2. The diffusion coefficient of 3–5 kDa dextran (103 µm^2 s^{-1}) in PBS was applied to the top and bottom compartments, while the fitted diffusion coefficient from the ***diffusion coefficient estimation*** model was applied to the hydrogel compartment. The top and hydrogel compartments had initial concentrations of 0 µM dextran, and the bottom compartment had an initial concentration of 3.2 µM dextran for all cases. All external boundaries were defined as no flux, unlike the internal boundaries (hydrogel interfaces with the top and bottom compartment) that allowed diffusion with the application of a flux continuity boundary condition. All COMSOL Multiphysics 5.4 simulations were solved using a direct MUMPS solver in the time-dependent study with an output range of 0 to 96 h time step of 0.5 h.

In order to simulate time interval changes in the dextran concentrations, an *Events* module was applied to the model in the top and bottom compartments. A *Discrete State* was added to the events with an initial value of 1, to specify the ON function. The $pulse_i$ variable was defined in the *Explicit Events* as a cyclical pattern with a period, T (where T was 12, 24 or 48 h). *Explicit Event-ON* started at an initial time of 0 h, and *Explicit Event-OFF* started at 1 h. The refreshment concentration was defined as 3.2 µM and 0 µM for the entire volume of bottom and top

compartments, respectively, using the pointwise constraint in the domains defined in equation S2.

$$Pointwise\ contraint = pulse_i * (Dextran_{i,t} - Dextran_{(i,t=0)}) \qquad \text{eq. S2}$$

Where $Dextran_{i,t=0}$ = initial dextran concentration in µM and $Dextran_{i,t}$ = dextran concentration at the current time point in the simulation in µM. The simulations were run with an "extra fine" mesh.

Case study 1. Bottom media change. Here, media time interval changes in the bottom compartment for every 12, 24, and 48 h was modeled by applying the *Event* module and pointwise constraint in the bottom compartment's domain (Figure 2b).

Case study 2. Media and PBS change. The media interval changes in the top and bottom compartments were modeled every 12, 24, and 48 h intervals by defining the *Events* in the top and bottom compartments' domains. In this case, we refreshed the top and bottom compartment with 3.2 µM concentration.

Case study 3. Concentration Burst modeled a concentration spike at 12 h in the bottom compartment, which is 0.5, 2 and 3-times the initial starting concentration at 0 h of the simulation. These spikes could be required when cells are proliferating or increasing their metabolism. For this case study, the *Events* variable spike was defined such that the *Explicit Event-ON* started at an initial time of 12 h with an infinite period, and *Explicit Event-OFF* started at 13 h with an infinite period. In addition, the pointwise constraint was adapted, as shown in equation S3.

$$Pointwise\ contraint_{Spike} = if\left(t < 12[h], \left(spike_i * \left(Dextran_{i,t} - Dextran_{i,t=0}\right)\right), \left(spike_i * \left(Dextran_{i,t} - \boldsymbol{f} * Dextran_{i,t=0}\right)\right)\right) \quad \text{eq. S3}$$

Where $Dextran_{i,t=0}$ = initial dextran concentration in µM and $Dextran_{i,t}$ = dextran concentration at the current time point in the simulation in µM, $spike_i$ is the event variable, and f is the concentration multiplier (f= 0.5, 2, 3).

Case study 4. Decay rate In the previous case studies, we assumed the growth factors did not degrade. However, many growth factors become less potent in cell culture, reflected in a decay rate. In case 4, we investigated the effect of a decay rate function on the cell culture growth factor concentration. The growth factors of interest were fibroblast growth factor 2 (FGF-2,$\tau_{1/2}$= 27 h)[14] and insulin-like growth factor 1 (IGF-1, $\tau_{1/2}$= 17 h)[15]. FGF-2 and IGF-1 were simulated using the estimated diffusion coefficient for 3–5 kDa since their molecular weights are similar[14–16], in particular in the medium a diffusion coefficient of 103 µm^2/s and in the hydrogel a diffusion coefficient of 48.39 µm^2/s was used. We modeled the cell culture scenario in which two common growth factors, FGF-2 and IGF-1, can decay using equation S4.

$$C_{t,i} = C_{0,i}e^{-\lambda_i t} \quad \text{eq. S4}$$

Where the growth factor is specified as i, the growth factor concentration at simulation time, t, is $C_{t,i}$ in µM, the initial concentration of the growth factor is $C_{0,i}$, in µM, the decay constant for growth factor i, is λ_i, in s^{-1}, and the simulation time, t in seconds.

The decay rate function for both growth factors was determined by calculating the derivative of equation S4:

$$\frac{dC_{t,i}}{dt} = -\lambda_i * C_{t,i} \quad \text{eq. S5}$$

Where the decay rate λ_i is $\frac{\ln(2)}{\tau_{1/2}}$ and $\tau_{1/2}$ is the half-life in seconds. We used as half-life of FGF2 and IGF-1 27h[14] and 17h[15], respectively. The decay rate function was applied to the top and bottom compartments using the *Reactions* domain with FGF2 and IGF-1 as the reacting species ($C_{t,FGF2}$ *and* $C_{t,IGF-1}$). FGF-2 and IGF-1 were simulated using the estimated diffusion coefficient for 3–5 kDa since their molecular weights are similar[14,15]. In this case, we refreshed the bottom compartment with 3.2 μM FGF-2 and IGF-1. The initial and refreshment concentrations were kept the same to the other cases, in order to be able to compare and investigate the effect of growth factor decay. However, the concentrations are similar to what is reported in literature for IGF-1 [2] and FGF-2 [3].

SUPPORTING FIGURES

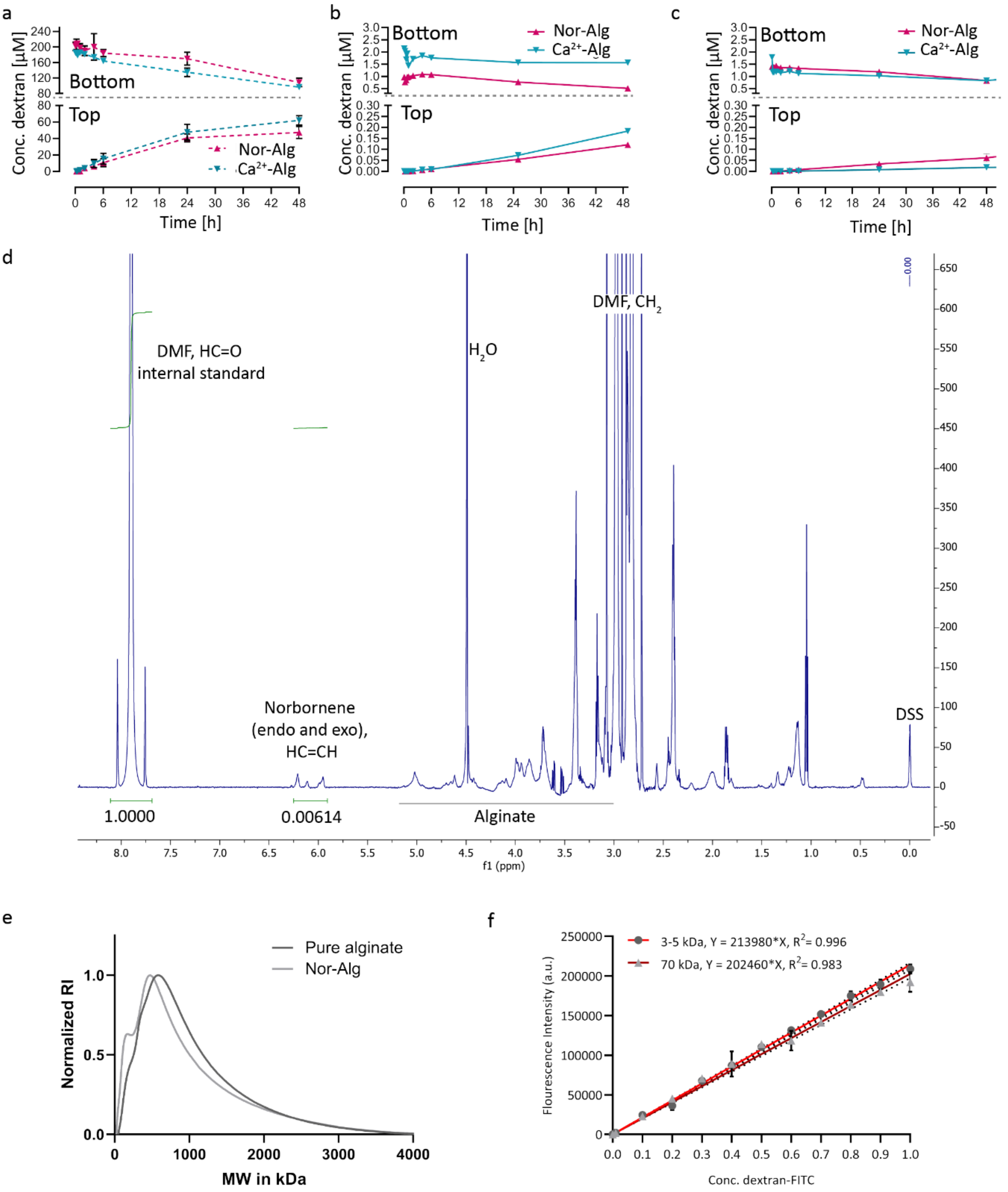

Figure S1. Diffusion time-series data, the concentration of a) 3–5 kDa dextran (on 0.2 µm pore size transwells, N=5) and b) 70 kDa dextran (on 8 µm pore size transwells, N=1) in the top and bottom compartment after diffusion through the hydrogels (Nor-Alg and Ca^{2+}-Alg). Change of transwell pore size was required as it impacts the diffusion through the whole system, as can be observed in c) where

concentration versus time data of 70 kDa Dextran in the top and bottom compartment after diffusion through the hydrogels (Nor-Alg and Ca^{2+}-Alg) compared to b). d) ^{1}H-NMR and e) GPC of norbornene functionalized alginate.[1] f) Standard curve used for diffusion time-series data fluorescence intensity to mg/mL dextran.

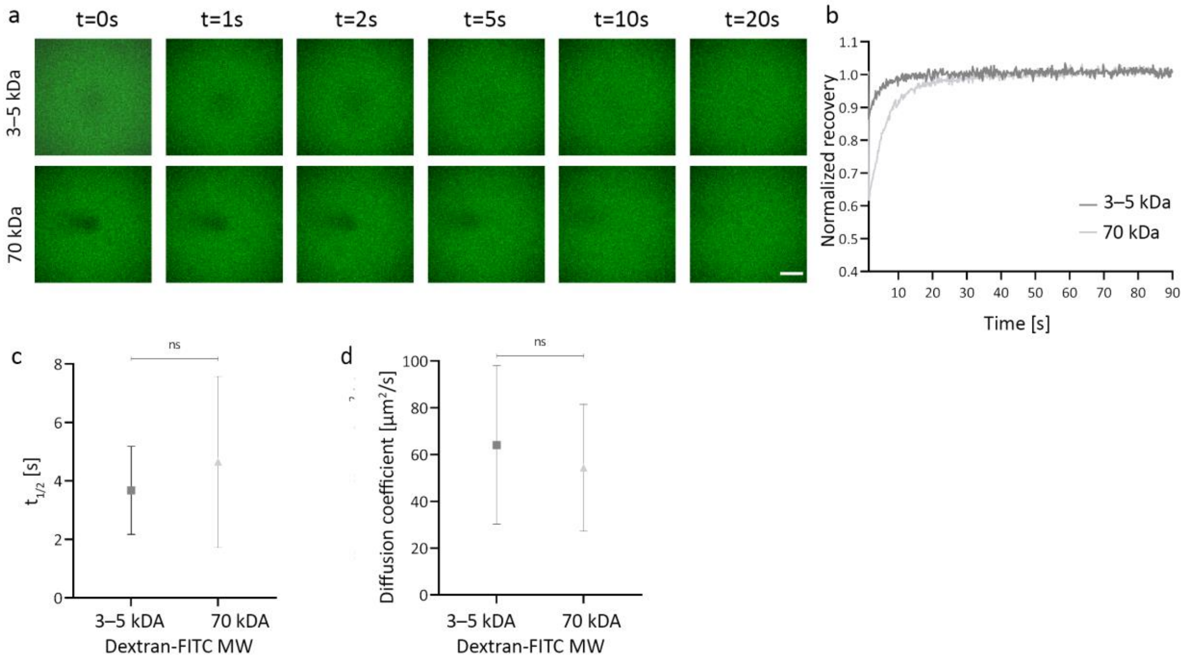

Figure S2. a) Fluorescence recovery after photobleaching (FRAP) images of 3–5 kDa dextran and 70 kDa from 0–60 s in PBS, and b) the corresponding recovery curves (n=3 difference bleaching areas, scale bar:100 µm). c) The half-time recovery ($t_{1/2}$) and d) diffusion coefficients were not significantly different (ns, one-way ANOVA) for either 3–5 or 70 kDa dextran in PBS.

[1] Same Nor-alg material batch as reported in Geuens & Ruiter (2021)[1] T. Geuens, F.A.A. Ruiter, A. Schumacher, F.L.C. Morgan, T. Rademakers, L.E. Wiersma, C.W. van den Berg, T.J. Rabelink, M.B. Baker, V.L.S. LaPointe, Thiol-ene cross-linked alginate hydrogel encapsulation modulates the extracellular matrix of kidney organoids by reducing abnormal type 1a1 collagen deposition, Biomaterials 275 (2021) 120976..

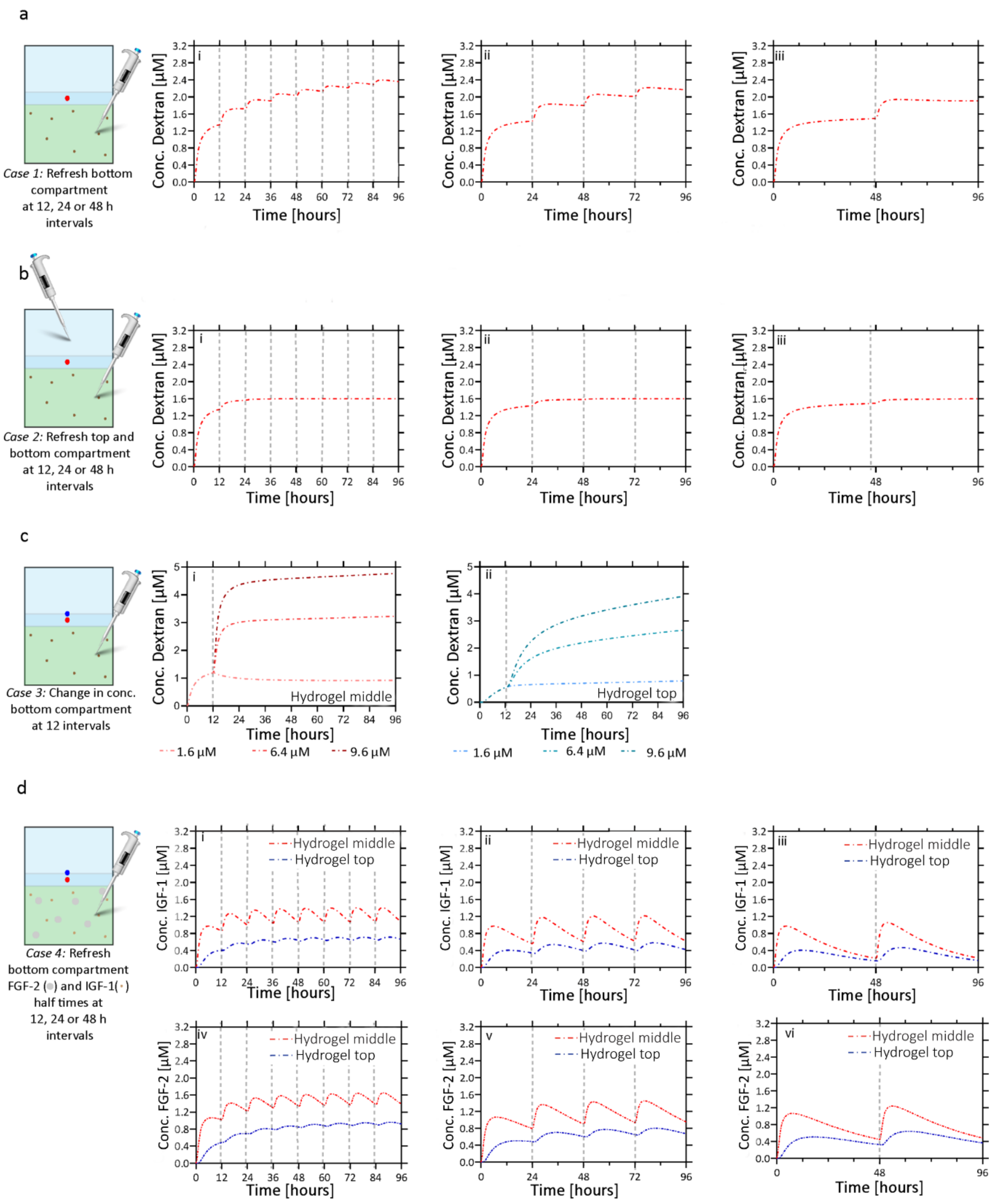

Figure S3. a) Case 1: the concentration (μM) of 3–5 kDa dextran at the middle of a 2 mm–thick Ca^{2+}-alg hydrogel was determined over time when the bottom compartment was refreshed with the starting dextran concentration of the bottom compartment (i.e. 3.2 μM) at i) 12, ii) 24 and iii) 48 h. b) Case 2: the concentration (μM) of 3–5 kDa dextran (μM) at the middle of a 2 mm–thick Ca^{2+}-alg hydrogel was determined over time when the top and bottom compartments were refreshed with the starting dextran

concentration of the bottom compartment (i.e. 3.2 µM) at i) 12, ii) 24 and iii) 48 h. c) Case 3: the concentration (µM) of 3–5 kDa was determined over time when the bottom compartment was refreshed with different concentrations (1.6, 6.4, and 9.6 µM) of 3–5 kDa after 12 hours of 3.2 µM conc. in the i) the middle of the 2 mm–thick hydrogel (red dot) and ii) at the top of the 2 mm–thick hydrogel (blue dot). d) Case 4: the concentration (µM) of IGF-1 (i-ii-iii) with a halftime 17h and FGF2 (iv-v-vi) with a halftime of 27h, respectively, at the middle (red), and top (blue) of a 2 mm–thick hydrogel were determined over time when the bottom compartment was refreshed at 12, 24 and 48 h with 3.2 µM. Grey vertical striped lines represent media refreshments points. The top and hydrogel compartments had initial concentrations of 0 µM dextran, and the bottom compartment had an initial concentration of 3.2 µM dextran for all cases. Figure S4 provides additional information on the concentration data at the top and bottom of the Ca^{2+}-alg hydrogel.

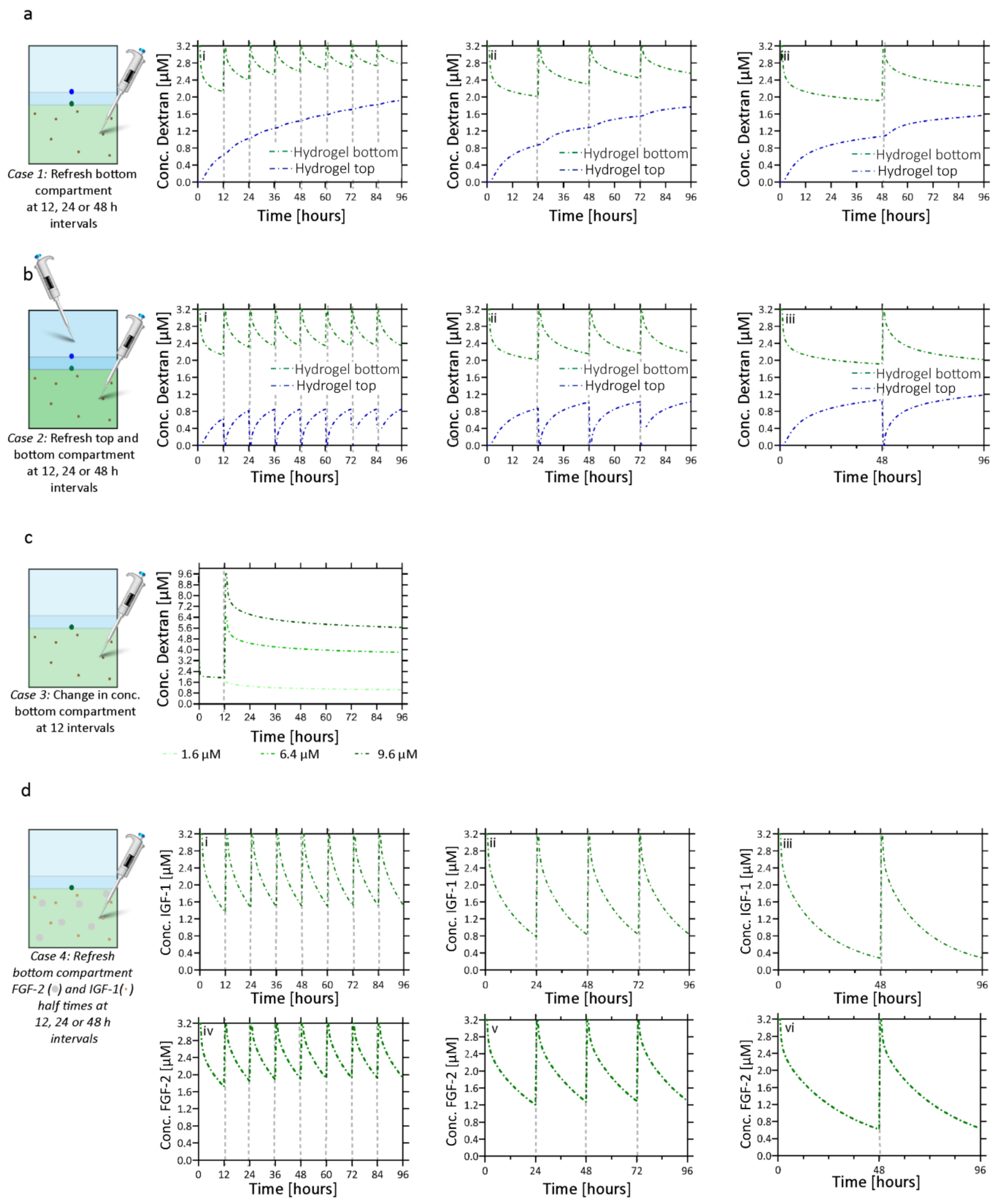

Figure S4. Concentration data for the Ca^{2+}-alg simulations at the top (blue) and bottom (green) of a 2 mm–thick Ca^{2+}-alg hydrogel. a) Case 1: the concentration (µM) of 3–5 kDa dextran at the top (blue) and bottom (green) of a 2 mm–thick Ca^{2+}-alg hydrogel was determined over time when the bottom compartment was refreshed with the starting dextran concentration of the bottom compartment (i.e. 3.2 µM) at i) 12, ii) 24 and iii) 48 h. b) Case 2: the concentration (µM) of 3–5 kDa dextran at the top (blue) and bottom (green) of a 2 mm–thick Ca^{2+}-alg hydrogel was determined over time when the top and

bottom compartments were refreshed with the starting dextran concentration of the bottom compartment (i.e. 3.2 µM) at i) 12, ii) 24 and iii) 48 h. c) Case 3: the concentration (µM) of 3–5 kDa dextran at the bottom of a 2 mm–thick hydrogel was determined over time when the bottom compartment was refreshed with different concentrations (1.6, 6.4, and 9.6 µM) of 3–5 kDa dextran after 12 hours of 3.2 µM conc. d) Case 4: the concentration (µM) of i-ii-iii) IGF-1 and iv-v-vi) FGF2 at the bottom of a 2 mm–thick hydrogel were determined over time when the bottom compartment was refreshed at 12, 24 and 48 h with 3.2 µM. Grey vertical stripped lines represent media refreshments points. The colors of the lines represent the sampling position. The top and hydrogel compartments had initial concentrations of 0 µM dextran, and the bottom compartment had an initial concentration of 3.2 µM dextran for all cases.

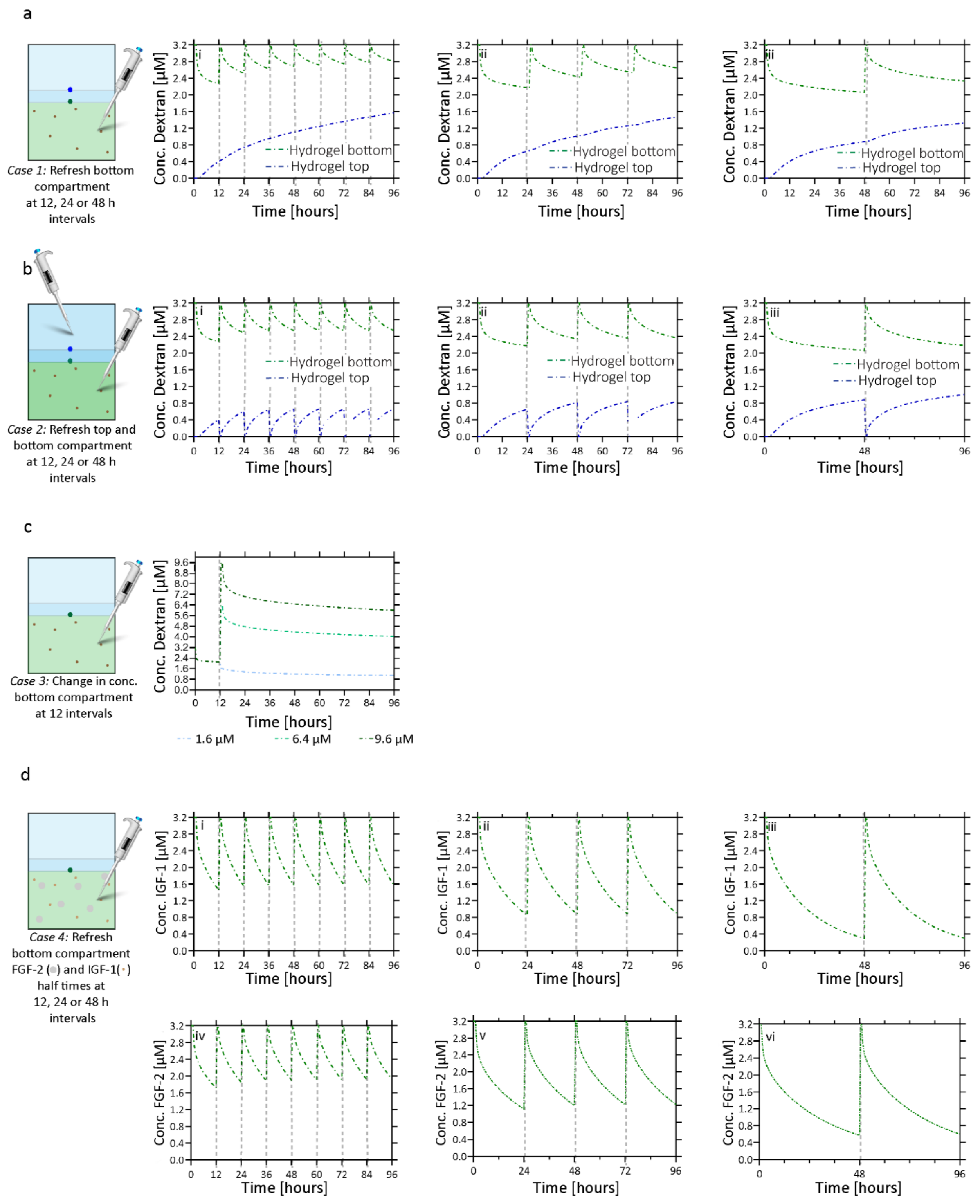

Figure S5. Concentration data for the Nor-alg simulations at the top (blue) and bottom (green) of a 2 mm–thick Nor-alg hydrogel (top and/or middle hydrogel concentrations represented in Figure 2). a) Case 1: the concentration (µM) of 3–5 kDa dextran at the top (blue) and bottom (green) of a 2 mm–thick Nor-alg hydrogel was determined over time when the bottom compartment was refreshed with the starting dextran concentration of the bottom compartment (i.e. 3.2 µM) at i) 12, ii) 24 and iii) 48 h. b)

Case 2: the concentration (µM) of 3–5 kDa dextran at the top (blue) and bottom (green) of a 2 mm–thick Nor-alg hydrogel was determined over time when the top and bottom compartments were refreshed with the starting dextran concentration of the bottom compartment (i.e. 3.2 µM) at i) 12, ii) 24 and iii) 48 h. c) Case 3: the concentration (µM) of 3–5 kDa dextran at the bottom of a 2 mm–thick hydrogel was determined over time when the bottom compartment was refreshed with different concentrations (1.6, 6.4, and 9.6 µM) of 3–5 kDa after 12 hours of 3.2 µM conc. d) Case 4: the concentration (µM) of i-ii-iii) IGF-1 and iv-v-vi) FGF2 with a half-time of 17h and 27h, respectively, at the bottom of a 2 mm–thick hydrogel were determined over time when the bottom compartment was refreshed at 12, 24 and 48 h with 3.2 µM. Grey vertical striped lines represents media refreshments points. The top and hydrogel compartments had initial concentrations of 0 µM dextran, and the bottom compartment had an initial concentration of 3.2 µM dextran for all cases.

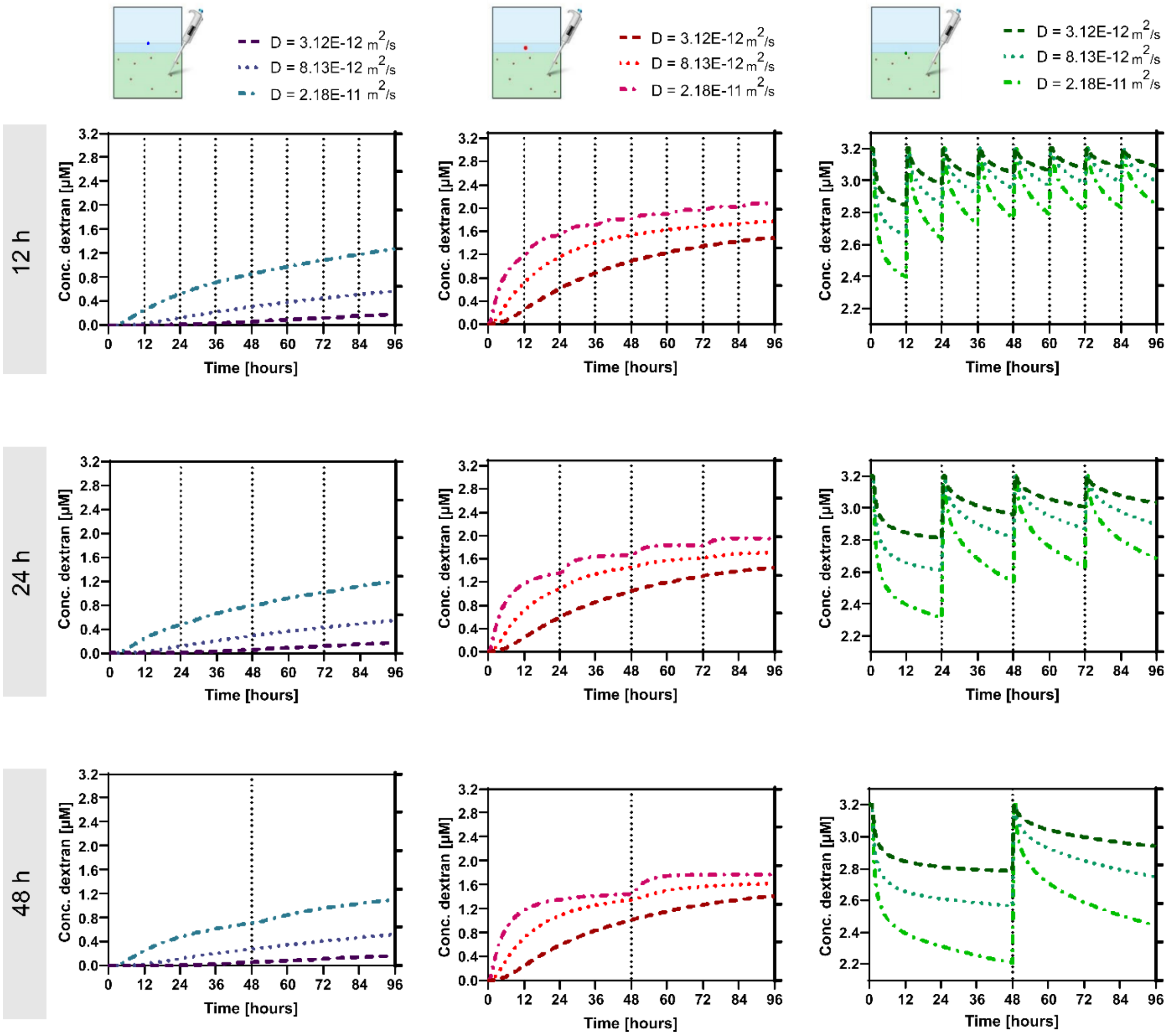

Figure S6. Concentration data for the Nor-alg simulations at three locations in a 2 mm–thick Nor-alg hydrogel for three different diffusion coefficients, based on [4]. The results focus here on Case 1 where the concentration (µM) of 3–5 kDa dextran at the top (blue), middle (red) or bottom (green) of a 2 mm–thick Nor-alg hydrogel was determined over time when the bottom compartment was refreshed with the starting dextran concentration of the bottom compartment (i.e. 3.2 µM) at 12h, 24h and 48 h. Grey vertical striped lines represents media refreshments points. The top and hydrogel compartments had initial concentrations of 0 µM dextran, and the bottom compartment had an initial concentration of 3.2 µM dextran for all cases.

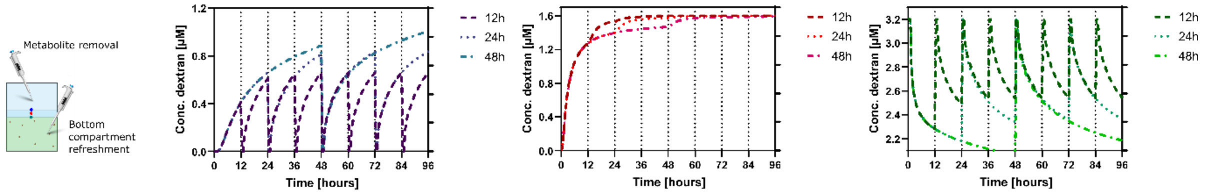

Figure S7. Concentration data for the Nor-alg simulations at three locations in a 2 mm–thick Nor-alg hydrogel for three different regimes of metabolite removal. The results focus here on Case 1 where the concentration (µM) of 3–5 kDa dextran at the top (blue), middle (red) or bottom (green) of a 2 mm–thick Nor-alg hydrogel was determined over time when the bottom compartment was refreshed with the starting dextran concentration of the bottom compartment (i.e. 3.2 µM) at 12h, 24h and 48 h and in the top compartment the dextran concentration was set to zero at 12h, 24h and 48h. Grey vertical striped lines represents media refreshments points. The top and hydrogel compartments had initial concentrations of 0 µM dextran, and the bottom compartment had an initial concentration of 3.2 µM dextran for all cases.

References

[1] T. Geuens, F.A.A. Ruiter, A. Schumacher, F.L.C. Morgan, T. Rademakers, L.E. Wiersma, C.W. van den Berg, T.J. Rabelink, M.B. Baker, V.L.S. LaPointe, Thiol-ene cross-linked alginate hydrogel encapsulation modulates the extracellular matrix of kidney organoids by reducing abnormal type 1a1 collagen deposition, Biomat. 275 (2021) 120976.

[2] Y.-l. Huang, R.-f. Qiu, W.-y. Mai, J. Kuang, X.-y. Cai, Y.-g. Dong, Y.-z. Hu, Y.-b. Song, A.-p. Cai, Z.-g. Jiang, Effects of insulin-like growth factor-1 on the properties of mesenchymal stem cells in vitro, J. Zhejiang Univ., Sci., B 13(1) (2012) 20-28.

[3] C.M. Kelly, R. Zietlow, S.B. Dunnett, A.E. Rosser, The Effects of Various Concentrations of FGF-2 on the Proliferation and Neuronal Yield of Murine Embryonic Neural Precursor Cells In Vitro, Cell Transplant. 12(3) (2003) 215-223.

[4] J.C. Breger, B. Fisher, R. Samy, S. Pollack, N.S. Wang, I. Isayeva, Synthesis of “click” alginate hydrogel capsules and comparison of their stability, water swelling, and diffusion properties with that of Ca+ 2 crosslinked alginate capsules, J. Biomed. Mater. Res., Part B 103(5) (2015) 1120-1132.
